# Stress Dependence of Seebeck Coefficient in Iron-Based Amorphous Ribbons

**DOI:** 10.3390/ma12172814

**Published:** 2019-09-02

**Authors:** Michał Nowicki

**Affiliations:** Warsaw University of Technology, Institute of Metrology and Biomedical Engineering, 02-525 Warsaw, Poland; nowicki@mchtr.pw.edu.pl; Tel.: +48-690-650-386

**Keywords:** Seebeck coefficient, amorphous ribbon, tensile stress, phonon drag, magnon drag

## Abstract

The results of an investigation on tensile stress dependence of mean Seebeck coefficient in Fe-based amorphous ribbons are presented, constituting a new Seebeck-sigma effect. A measurement test stand, capable of the determination of small variations in thermopower in such materials under stress is described. Exemplary results for commercially available, positively magnetostrictive SA1 and 2605CO amorphous ribbons show significant stress dependence with more than 1% of relative change, in contrast to negatively magnetostrictive 6030D alloys with 0.1% change. Non-ferromagnetic alloys are tested for comparison purposes, giving negligible results. Thus, the possibility of a magnetomechanical mechanism of the stress influence is proposed.

## 1. Introduction

Since the discovery of amorphous alloys, there is a massive amount of ongoing research devoted to their physical properties, as well as various effects enabling their utilization, mostly based on unique mechanical and magnetic properties [1,2,3,4,5,6,7]. Among the many magnetic-related phenomena, one of the more interesting, and relatively under-researched are the various magnetomechanical effects [8], with magnetostriction and inverse magnetomechanical (Villari) effects [9] as two main examples. These magnetomechanical effects can be measured with very high precision, e.g., by using piezo and optical [10,11,12] methods. However, the great list of magnetomechanical effects given by Williams [13] hints at “change of thermal EMF (electromotive force) due to magnetization” as another magnetomechanical effect. This is understandable, as the various electronic properties of metals are closely interconnected. Reports of conducting thermoelectric research on magnetic amorphous ribbons are uncommon [14,15,16,17,18]. There also seem to be no published results of the effects of magnetic field on the Seebeck coefficient in amorphous ferromagnetic metals. Given that amorphous alloys magnetic properties can have significant stress sensitivity [19], this paper aims at presenting first results of tensile stress influence on the Seebeck coefficient in commercially-available Fe-based amorphous ribbons, constituting the Seebeck-sigma effect.

## 2. Materials and Methods

### 2.1. Utilized Samples

The ribbon samples used in the investigation were made from commercially available, amorphous alloys listed in Table 1. Fe_80_B_11_Si_9_ and Fe_40_Co_38_Sio_4_B_18_ alloys were chosen due to their significant positive magnetostriction, and Co_84_Fe_1.5_Mo_2_Mn_1.5_Si_7_B_2_ due to smaller negative magnetostriction. Furthermore, typical thermoelectric constantan and chromel alloys were investigated for comparison purposes and for a test stand check. The Seebeck coefficients relative to the copper obtained from them are consistent with published data [20], which validates its accuracy. 

### 2.2. Measurement Method

**Figure 1:**
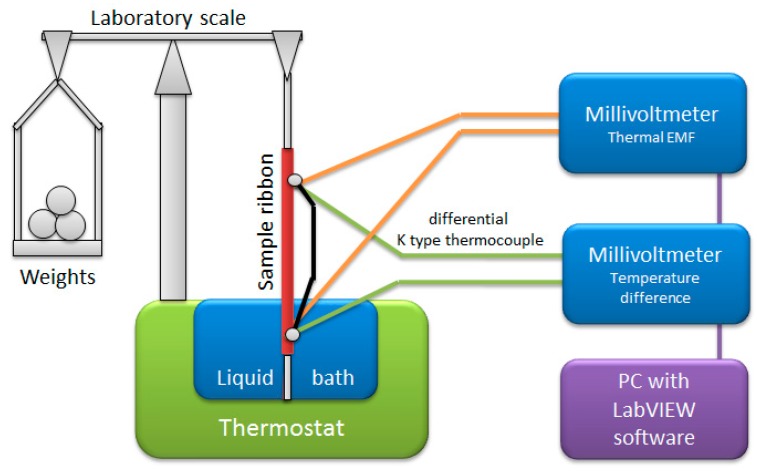
Schematic diagram of the developed Seebeck (σ) system.

The measurements were carried out on the specially designed, personal computer-controlled measurement system. Figure 1 presents the schematic diagram of this system.

The sample ribbons were mounted with one end (hot thermocouple junction) in a thermostat controlled liquid bath, and the other in free air, with additional styrofoam insulation (cold junction). The connections from hot and cold junctions were made of pure copper wire, connected to digital millivoltmeter for thermal EMF measurement. Additionally, a second millivoltmeter equipped with differential K-type thermocouple was used for the temperature difference measurement. The PC equipped with the LabVIEW developed program allowed for simultaneous recording of temperature difference, thermopower of the sample against the copper and calculation of mean Seebeck coefficient [21]. Due to near-linear characteristics of most metals (for small temperature variations), the relative Seebeck coefficient of the sample was calculated simply, as:

(1) S=VTΔT

where *S* is the Seebeck coefficient of the sample relative to copper, in μV/K, *V_T_* is the measured thermoelectric voltage in μV, Δ*T* is the temperature difference in K between the hot and cold junctions of the sample. The obtained value of *S* is not exactly the Seebeck coefficient of the given material, as it ideally should be measured for very small temperature differences. However, the value obtained in the presented way is near-constant for ±10% temperature variations of the hot junction. Thus, operation on this coefficient ensures greater independence from fluctuations in the set temperature than operation on *V_T_* alone. What is more, the use of dedicated software speeds up the measurements, and fast repeating of readings allows for the averaging and filtering of the results (program was set to filter and average 1000 measurements). The system allows for discrimination of changes in the Seebeck coefficient at the 0.05% level. To obtain this level of accuracy with utilized voltmeters, Δ*T* was set to 100 °C above ambient temperature. Otherwise, nanovoltmeters and sufficient shielding should be used.

Tensile stress was generated in the samples by means of equal-arms laboratory scale and weights. This approach allowed for high-precision of tensile force application, as well as negligible force offset. Therefore, the only non-negligible component of the stress uncertainty was the measurement of the sample cross-section, which can be approximated as less than 1%.

## 3. Results and Discussion

**Figure 2:**
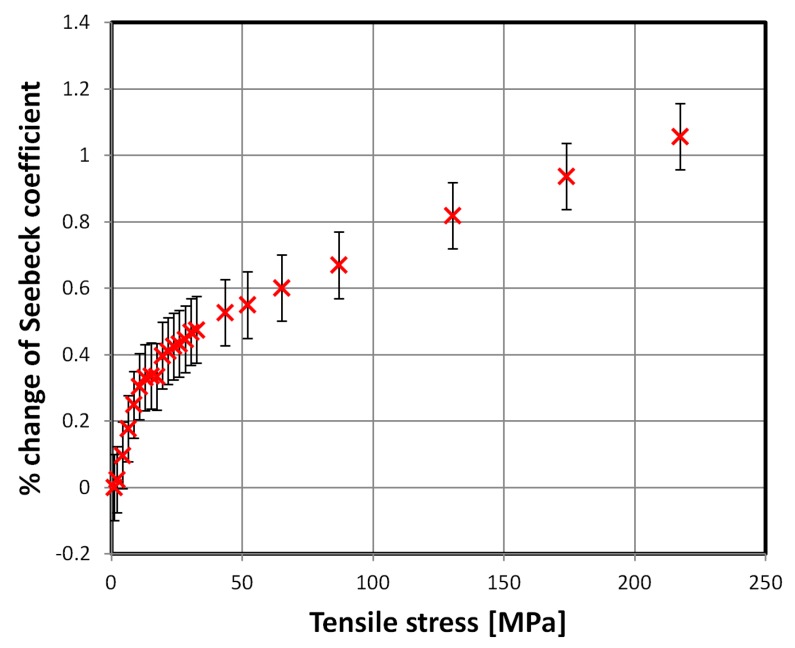
Relative change of the mean Seebeck coefficient due to tensile stresses, SA1 alloy.

**Figure 3:**
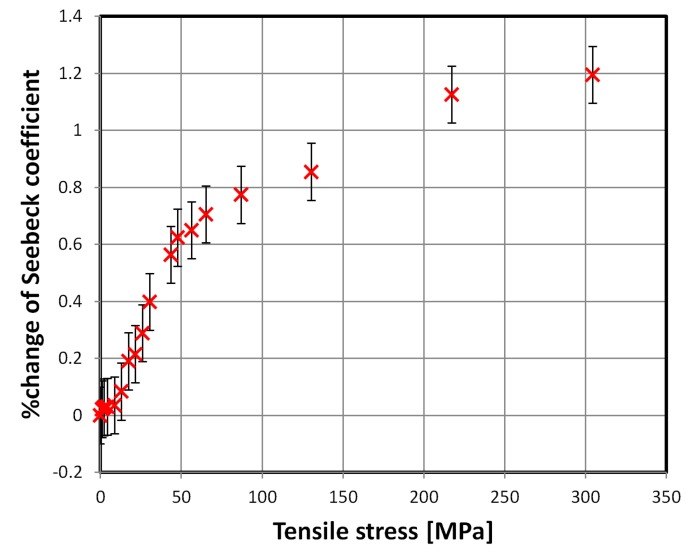
Relative change of the mean Seebeck coefficient due to tensile stresses, 2605CO alloy.

First two of the investigated materials exhibited significant influence of tensile stress on the mean Seebeck coefficient. The results for SA1 and 2605CO alloys are presented in Figure 2 and Figure 3, respectively.

The 6030 alloy exhibited maximum relative change in *S* of 0.1%, with local maximum between 50 and 150 MPa, and a rapid decrease for higher stresses; however, the uncertainty of the measurements was comparable to the effect, and was thus too high to present reliable characteristics.

Constantan shows a repeatable drop of the *S* coefficient of about 0.05% for rapid application of 100 MPa of tensile stress; obtaining of the full characteristic, however, was also hindered by the signal noise. For Chromel, typically used in K-type thermocouples, a similar effect was observed.

The characteristics were measured in 400 K temperature. Further studies will be needed to investigate the temperature dependence of the observed effect.

As the Seebeck coefficient is largely determined by various electron-scattering mechanisms, such as phonon drag [20], it may be proposed that the overall change observable in presented results is due to varying phonon-electron interactions, or another mechanism influencing the mean free path of the electrons.

The stress-Seebeck characteristic of the SA1 alloy, presented in Figure 2, has two distinct regions. First, with faster changes, up to about 20 MPa of tensile stress, and later with an almost linear, slower rise. The first region corresponds in applied stress span with high magnetoelastic sensitivity region of this alloy. The same effect is observable for the 2605CO alloy up to 50 MPa of tensile stress. Thus, change of stress-induced magnetoelastic energy in the investigated materials, is given by Equation (2). The changing balance of total energy density *E*, may be responsible for the observed behavior.

(2) $$E_{M}=\frac{3}{2}\lambda_{S}\sigma$$

There may also be a varying level of magnon drag present, which is one of the Seebeck coefficient’s determining mechanisms in ferromagnetic metals [22]. The effect is relatively weak, constituting up to 0.5% of Seebeck coefficient of Fe and Ni [23], and up to 5% in Gd [24]. This effect peaks in a lower temperature region than investigated in this paper (around 200 K for Fe). However, given the measured fast-rise changes are in the order of 0.2%, it is a possibility.

It is especially interesting, that the stress induced change in thermopower is at least one order of magnitude greater for positively magnetostrictive amorphous ribbons, compared to other investigated materials.

## 4. Conclusions

The measurement stand capable of measurements of tensile stress influence on thermopower of ribbon samples was presented. Exemplary results of investigation of positively magnetostrictive amorphous ribbons are given for the first time, presenting the new Seebeck-sigma effect. The effect is an order of magnitude greater than in negative-magnetostrictive samples. Possible causes responsible for the observed behavior are proposed. However, further multiphysical studies are needed to quantify the effect, which may prove interesting for the investigation of electronic effects in amorphous metals. Furthermore, the measurement methods will need to be substantially refined, to reliably quantify this effect in non-ferromagnetic samples.

In order to physically model the observed effect, thermodynamical analysis should be performed, to account for the interplay between magnetostricition, strain, and thermoelectric properties. The thermodynamics of irreversible processes which describe magnetoelectric coupling could serve as a base for such modeling, however, much more additional data would need to be obtained, such as the magnetic field effect on thermopower, temperature dependence of the S(σ) characteristics, and magnetoelastic characteristics of the investigated amorphous alloys.

## Figures and Tables

**Table 1:** Essential parameters of investigated samples.

Manufacturer and Trade Name	Chemical Composition	Maximal Permeability in As-Cast State μ	Magneto-Striction in Saturation λ_s_ (μm/m)	Saturation Induction B_s_ (T)	Seebeck Coefficient Relative to Copper (µV/K) ^1^
Metglas SA1	Fe_80_B_11_Si_9_	45,000	27	1.56	7.35
Metglas 2605CO	Fe_40_Co_38_Sio_4_B_18_	150,000	35	1.8	7.32
Vacuum-schmelze 6030 D30	Co_84_Fe_1.5_Mo_2_Mn_1.5_Si_7_B_2_	450,000	−11.8	0.82	6.86
Constantan	Cu_55_Ni_45_	N/A	N/A	N/A	−42.7
Chromel	Ni_90_Cr_10_	N/A	N/A	N/A	15.2

^1^ Results taken from the presented test stand. For standardized Seebeck coefficient relative to platinum add 6.5.
